# Clinical and immunological analysis of letermovir for preventing cytomegalovirus infection in children after hematopoietic stem cell transplantation

**DOI:** 10.3389/fimmu.2026.1817638

**Published:** 2026-04-22

**Authors:** Qing Liu, Bing Zhou, Na Zhang, Hong Li, Sijian Wang, Shumin Hou, Yilin Wang, Pan Fu, Tianqiao Zhong, Jingbo Shao, Kai Chen, Hui Jiang

**Affiliations:** 1 Department of Hematology and Oncology, Shanghai Children’s Hospital, School of Medicine, Shanghai Jiao Tong University, Shanghai, China; 2 Clinical Laboratory Department, Shanghai Children's Hospital, School of Medicine, Shanghai Jiao Tong University, Shanghai, China

**Keywords:** children, cytomegalovirus, hematopoietic stem cell transplantation, immune reconstitution, letermovir, prevention

## Abstract

**Objective:**

The present study aimed to analyze the efficacy and safety of letermovir for preventing cytomegalovirus (CMV) infection and its impact on immune reconstitution in children after allogeneic hematopoietic stem cell transplantation (allo-HSCT).

**Methods:**

The retrospective analysis included 83 patients who underwent allo-HSCT at Shanghai Children’s Hospital between January 2022 and June 2025 and survived ≥100 days post-transplantation. Forty patients received letermovir as primary CMV prophylaxis (letermovir group), and 43 patients did not receive prophylaxis (control group). The primary outcomes were the post-transplantation incidence of CMV infection and CMV disease. Secondary outcomes included the effects of letermovir on acute graft-versus-host disease (aGVHD), hematopoietic reconstitution (time to neutrophil and platelet engraftment), liver function, incidences of Epstein-Barr virus (EBV) and BK virus (BKV) infections, and cellular immune reconstitution.

**Results:**

The cumulative incidence of CMV infection post-transplantation was significantly lower in the letermovir group than in the control group (100 days: 7.5% vs. 53.5%, p<0.001; 180 days: 12.5% vs. 53.5%, p<0.001). No CMV disease occurred in either group. CMV reactivation occurred in 3 patients (7.5%) after discontinuation of letermovir. In the letermovir group, CMV infection occurred later [mean (range): 90.7 (31–168) days vs. 42.7 (16–90) days, p=0.050], lasted for a shorter duration [11.8 (7–15) days vs. 17.5 (4–39) days, p=0.042], and had a lower mean viral load [3,175 copies/ml vs. 30,407 copies/ml, p=0.393]. No significant differences were observed between the two groups in the time to neutrophil and platelet engraftment, incidence of grade II–IV and III–IV aGVHD, incidence of liver function abnormalities, or rates of EBV/BKV infection. Multivariate analysis showed that letermovir prophylaxis was an independent protective factor against CMV infection within 100 days post-transplantation (hazard ratio: 0.127, p=0.001). At 90 days, 180 days, and 1 year post-transplantation, the T lymphocyte and B lymphocyte counts were significantly lower in the letermovir group than in the control group (p<0.05). By 1 year post-transplantation, there was no difference in NK cell counts between the two groups (p=0.094). Within the letermovir group, only CD8^+^ T cell counts were significantly increased 1 year post-transplantation compared to pre-transplantation (p=0.027).

**Conclusion:**

Letermovir is safe and effective at reducing the risk of CMV infection in children after allo-HSCT, significantly delaying the onset of infection and shortening its duration. Although letermovir may impact early lymphocyte subset reconstitution, immune function can recover to pre-transplantation levels by 1 year post-transplantation.

## 1 Introduction

Cytomegalovirus (CMV) infection is one of the most common complications following allogeneic hematopoietic stem cell transplantation (allo-HSCT), particularly in pediatric patients. Due to children’s immature immune systems, the infection rate in CMV-seropositive recipients can reach 40-60%, significantly increasing transplant-related mortality (1, 2). Although current prophylactic agents, such as ganciclovir, valganciclovir, and foscarnet, are somewhat effective, their use is limited by the risks of myelosuppression and nephrotoxicity in the pediatric population. These risks are particularly concerning for children with delayed platelet recovery or renal impairment (3, 4). Letermovir, as a novel inhibitor of the CMV terminase complex, has demonstrated excellent efficacy in CMV prevention in adults and adolescents due to its unique mechanism of action and favorable safety profile (5, 6). However, data on its efficacy and safety in children under 12 years of age are relatively scarce, and its long-term impact on immune reconstitution post-transplantation is still not well-defined.

This study retrospectively analyzed the clinical data of 83 pediatric patients who underwent allo-HSCT at our center. The focus was on evaluating the efficacy and safety of letermovir for CMV prophylaxis and preliminarily exploring its effects on immune reconstitution. The aim was to provide evidence for the clinical application of letermovir in pediatric patients.

## 2 Methods

### 2.1 Patients

This study retrospectively included 104 children who underwent allo-HSCT at Shanghai Children’s Hospital from January 2022 to June 2025. Starting from August 2023, our center began using letermovir for the prevention of CMV infection post-transplantation. The medication was initiated within 30 days after transplantation and continued until 100 days post-transplantation. The following exclusion criteria were applied: death within 100 days post-transplantation, receipt of a second transplant, use of letermovir for secondary prophylaxis, and refusal to use letermovir due to family-related reasons. A total of 21 cases were excluded, and 83 patients were ultimately included in the analysis. Based on the prophylactic regimen, 40 patients who received letermovir as primary prophylaxis between August 2023 and June 2025 were assigned to the letermovir group, and 43 patients who did not receive letermovir between January 2022 and July 2023 served as the control group. The study conformed to institutional guidelines and was approved by the Institutional Review Board and local ethics committees. All procedures complied with the Declaration of Helsinki and relevant ethical standards for human research.

### 2.2 Transplantation protocol

The preconditioning regimen was determined based on the nature of the underlying disease. For patients with non-malignant disease, the protocol consisted of fludarabine (Flu), busulfan (Bu), cyclophosphamide (Cy), and rabbit anti-human thymocyte globulin (ATG). Considering the day of allo-HSCT as day 0, Flu (40 mg/m²/day) was administered intravenously on days -9 to -6, Bu (0.8 mg/kg/dose) intravenously every 6 hours on days -8 to -6, Cy (50 mg/kg/day) intravenously on days -5 to -4, and ATG (7.5 mg/kg) on days -4 to -2. For patients with malignant disease, the protocol consisted of Flu, Bu, thiotepa (TT), melphalan (Mel), and ATG. Flu (40 mg/m²/day) was administered intravenously on days -9 to -6, Bu (0.8 mg/kg/dose) intravenously every 6 hours on days -8 to -7, TT (10 mg/kg/day) intravenously on day -6, Mel (70 mg/m²/day) intravenously on days -5 to -4, and ATG (7.5 mg/kg) on days -4 to -2.

### 2.3 CMV prevention and monitoring

All patients received ganciclovir prophylaxis during the preconditioning period (days -9 to -1) at a dose of 10 mg/kg/day. Patients in the letermovir group initiated letermovir prophylaxis within 30 days post-transplantation. The prophylactic dose was determined by body weight: ≥30 kg, 480 mg/day; 15–30 kg, 240 mg/day; 7.5–15 kg, 120 mg/day. The dose was halved for patients receiving cyclosporine for graft-versus-host disease (GVHD) prophylaxis. The specific initiation time was determined by the treating physician based on clinical circumstances. In the control group, no letermovir or other routine CMV prophylaxis was administered after transplantation. Patients in both groups received oral acyclovir (10 mg/kg, twice daily) for herpesvirus prophylaxis from day 1. The CMV-DNA load was detected in whole blood and plasma samples using quantitative polymerase chain reaction (qPCR). The monitoring frequency was twice weekly before neutrophil engraftment and once weekly from engraftment to day 100; thereafter, regular monitoring was performed during outpatient follow-up. The diagnostic criteria for CMV viremia were from the literature (7). Preemptive antiviral therapy was initiated upon two consecutive CMV-DNA loads >500 copies/ml or a single result >1000 copies/ml. Preemptive therapy was initiated with either ganciclovir or foscarnet and was discontinued after two consecutive negative CMV-DNA tests.

### 2.4 Immune function monitoring

Basic lymphocyte subpopulations were analyzed using a FACSCalibur flow cytometer (BD Biosciences) and reported as absolute counts. FCM with CellQuest software (BD Biosciences) was used for analysis of lymphocyte subsets (CD3/CD45/CD4/CD8/CD16CD56/CD19, BD Biosciences), including T cells (CD3+CD45+), cytotoxic T cells (CD3+CD8+CD45+), helper T cells (CD3+CD4+CD45+), NK cells (CD16+CD56+CD3−CD45+), and B cells (CD19+CD45+). A total of 15,000 lymphocytes were acquired for analysis. Data were collected at baseline and on 90 days, 180 days, and 1year (post-transplantation).In the letermovir group, 17 patients had less than 1 year of post-transplant follow-up and did not undergo lymphocyte subpopulations monitoring 1 year post-transplantation.

### 2.5 GVHD prevention and treatment

Diagnosis and grading of acute and chronic GVHD were based on internationally recognized criteria (8–11). All children received cyclosporine, mycophenolate mofetil (MMF), and short-course methotrexate (MTX) for GVHD prophylaxis(tacrolimus was used for patients intolerant to cyclosporine). In case of acute GVHD (aGVHD), methylprednisolone (1–2 mg/kg/day) was administered. For steroid-refractory aGVHD, other options were considered, including basiliximab, tacrolimus, MMF, MTX, or mesenchymal stem cell infusion.

### 2.6 Definitions and evaluation of efficacy

Granulocyte engraftment was defined as the first of 3 consecutive days with an absolute neutrophil count >0.5×10^9^/L. Platelet engraftment was defined as the first of seven consecutive days with a platelet count >20×10^9^/L without transfusion support. Primary graft failure was a failure to achieve granulocyte engraftment by day 28. Secondary graft failure was a recurrence of persistent absolute neutrophil count <0.5×10^9^/L after initial hematopoietic reconstitution. Overall survival was the time from hematopoietic stem cell infusion to the end of follow-up or death.

### 2.7 Statistical analysis

All data were analyzed using SPSS 24.0 (IBM) statistical software. The clinical characteristics of the patients were presented using descriptive statistical methods; continuous variables were expressed as the median and range or mean ± standard deviation(SD), whereas categorical variables were expressed as frequencies (number and percentage). Comparisons of continuous variables between groups were performed using t-tests, and comparisons of categorical variables were performed using Pearson χ² tests or Fisher exact tests.

The cumulative incidence curve of CMV infection was plotted using the Kaplan–Meier method and compared using the log-rank test. Factors influencing CMV infection after transplantation were analyzed using the Cox proportional hazards regression model. A p-value < 0.05 was considered significant. All figures were generated using GraphPad Prism software.

## 3 Results

### 3.1 Clinical characteristics of patients

A total of 83 pediatric hematology patients with a median age of 9.6 years (range: 1.6–16 years) were included in this study; 40 cases were in the letermovir group and 43 in the control group. No significant differences were observed between the two groups in regard to baseline clinical characteristics or transplantation-related features (**Table 1**). In the letermovir group, the median time to initiation of prophylaxis after transplantation was 16 days (range: 1–34 days), and all patients continued medication until 100 days post-transplantation. Subgroup analyses of the letermovir group found that the mean time to initiation of prophylaxis in CMV-DNA-positive patients (23 ± 7.87 days) was later than the time in CMV-DNA-negative patients (16.54 ± 7.2 days), but the difference did not reach significance (t=1.856, p=0.071). The mean prophylactic doses were similar between the two subgroups: 8.57 ± 3.83 mg/kg in the positive group and 8.02 ± 2.9 mg/kg in the negative group (t=0.379, p=0.707).

**Table 1:** Baseline characteristics of the patients.

Characteristic		Letermovir group	Control group	P-value
n		40	43	
Sex, n (%)				0.991
	female	14 (35)	15 (34.9)	
	male	26 (65)	28 (65.1)	
Age, years				
	mean ± SD	9.76 ± 3.72	8.76 ± 3.22	0.197
Primary reason for transplantation, n (%)				0.308
	benign disease	24 (60)	21 (48.8)	
	malignant disease	16 (40)	22 (51.2)	
CMV antibody combination, n (%)				0.351
	donor+/recipient+	37 (92.5)	34 (79.1)	
	donor+/recipient−	2 (5)	4 (9.3)	
	donor−/recipient+	1 (2.5)	4 (9.3)	
	donor−/recipient−	0 (0)	1 (2.3)	
Type of donor, n (%)				0.384
	MSD	3 (7.5)	5 (11.6)	
	MUD	15 (37.5)	10 (23.3)	
	HID	22 (55)	28 (65.1)	
Drugs used for GVHD prophylaxis, n (%)				1
	cyclosporine based	36 (90)	39 (90.7)	
	tacrolimus based	4 (10)	4 (9.3)	
MNC, 10^8^ cells/kg				
	mean ± SD	12.26 ± 4.0	14.06 ± 5.10	0.075
CD34, 10^6^ cells/kg				
	mean ± SD	5.91 ± 1.83	5.01 ± 3.18	0.117
ABO match graft, n (%)				0.429
	matched	17 (42.5)	22 (51.2)	
	mismatched	23 (57.5)	21 (48.8)	
Conditioning regimen, n (%)				0.762
	MAC	21 (52.5)	24 (55.8)	
	RIC	19 (47.5)	19 (44.2)	

MSD, matched sibling donor; MUD, matched unrelated donor; HID, haploidentical donor; MNC, mononuclear cell; MAC, myeloablative transplantation; RIC, reduced intensity conditioning.

### 3.2 CMV infection status

**Figure 1:**
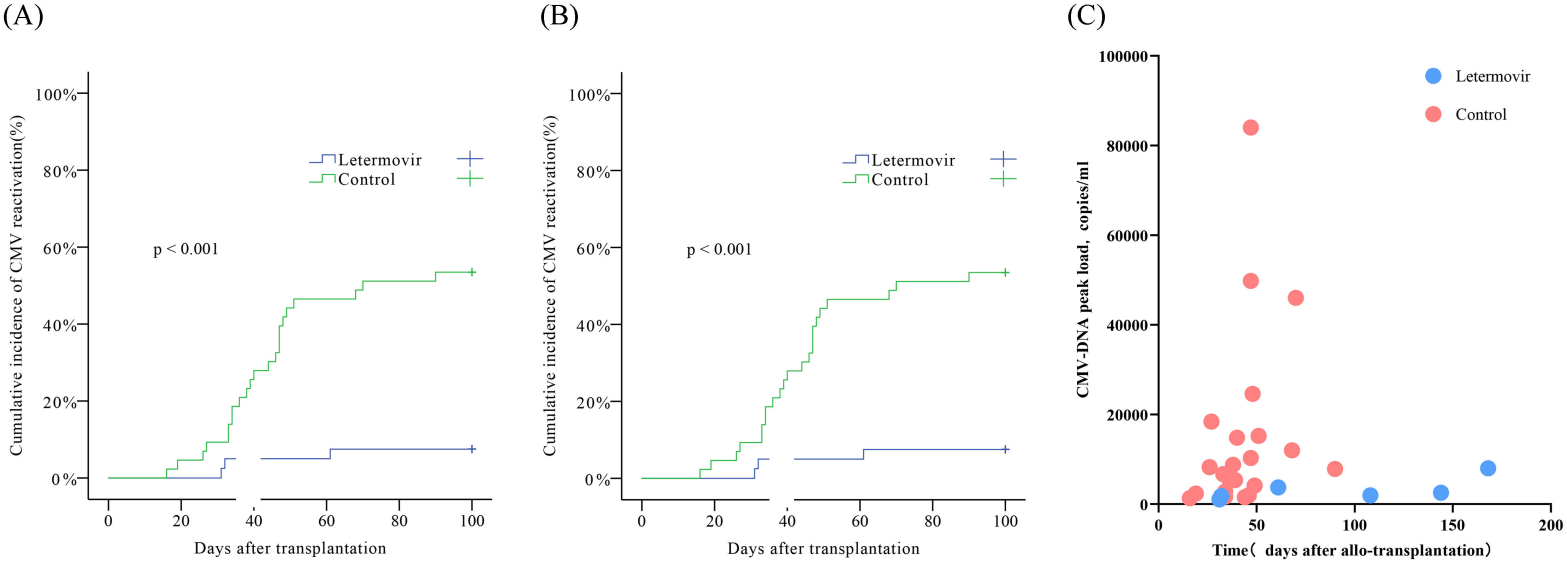
Incidence of CMV infection. **(A)** Cumulative incidence of CMV infection within 100 days **(B)** and 180 days post-transplantation. **(C)** Scatter plot showing the timing of infection onset and peak CMV-DNA levels.

Within 100 days after transplantation, the cumulative incidence of CMV infection in the letermovir group was 7.5% (n=3), which was significantly lower (χ²=18.538, p<0.001) than the incidence in the control group (53.5%, n=23; **Figure 1A**). Between 100 and 180 days after transplantation, three patients in the letermovir group experienced CMV reactivation on day 108, 144, and 168. One of these patients had a previous CMV activation within the first 100 days and was complicated with grade III intestinal GVHD. No new cases of CMV activation were observed in the control group during this period. By 180 days post-transplantation, the cumulative incidence of CMV viremia in the letermovir group was 12.5% (n=5), which was still significantly lower (χ²=18.538, p<0.001) than the incidence in the control group (**Figure 1B**). No CMV disease occurred in either group. Compared with the control group, the letermovir group had a significantly delayed mean time to CMV infection [90.7 (range: 31–168) days vs. 42.7 (range: 16–90) days; t=1.994, p=0.050] and a significantly shorter mean duration of infection [11.8 (range: 7–15) days vs. 17.5 (range: 4–39) days; t=2.144, p=0.042]. In addition, the mean CMV-DNA load in the letermovir group was lower [3,175 (range: 1,050–7,990) copies/ml vs. 30,407 (range: 1,330–366,000) copies/ml], but the difference did not reach significance (t=−0.868, p=0.393; **Figure 1C**).

### 3.3 Clinical outcomes after transplantation

We did not find significant differences between the letermovir group and control group in terms of the time to neutrophil engraftment or platelet engraftment (**Table 2**). The incidence and severity of aGVHD and the incidence of impaired liver function did not differ significantly between the two groups. Although the letermovir group had numerically lower rates of EBV infection (52.5% vs. 65.1%) and BKV infection (22.5% vs. 34.9%) compared with the control group, these differences did not reach significance (**Table 2**). Furthermore, no patient in the letermovir group discontinued the medication due to drug-related adverse events.

**Table 2:** Comparison of transplantation outcomes between the two groups of patients.

Outcome	Letermovir group	Control group	P-value
Neutrophil engraftment, Median,(rang),days	12 (10-19)	11 (9-15)	0.161
Platelet engraftment, Median,(rang),days	14 (7-27)	13 (9-25)	0.343
Acute GVHD,n(%)	13 (32.5)	22 (51.2)	0.085
Grade II;-IV,n(%)	5 (12.5)	11 (25.6)	0.131
Grade III-IV,n(%)	3 (7.5)	6 (14)	0.485
Hepatic impairment,n(%)	8 (20)	13 (30.2)	0.284
EBV,n(%)	21 (52.5)	28 (65.1)	0.243
BKV,n(%)	9 (22.5)	15 (34.9)	0.214
CMV,n(%)	5 (12.5)	23 (53.5)	0.000

### 3.4 Analysis of risk factors for CMV infection

Univariate Cox regression analyses indicated that grade II-IV aGVHD and the use of letermovir for prophylaxis were factors associated with CMV infection within 100 days after transplantation (**Table 3**). Multivariate Cox regression analysis demonstrated that the use of letermovir for prophylaxis was an independent protective factor against CMV infection within 100 days after transplantation (**Table 4**).

**Table 3:** Univariate Cox regression analysis of clinically significant CMV infection(csCMVi)during 100 days after HSCT.

Variable	Subcategory	HR	95% CI	P-value
Age		0.960	0.873-1.056	0.400
Sex	male/female	1.611	0.745-3.485	0.226
Primary reason for transplantation	benign/malignant disease	01.029	0.476-2.224	0.943
CMV antibody combination	donor+/recipient+			0.692
	donor+/recipient−	0.975	0.228-4.161	0.973
	donor−/recipient+	2.094	0.624-7.027	0.232
	donor−/recipient−	0.000	0.000-0.000	0.979
Type of donor	MSD			0.549
	MUD	2.768	0.346-22.135	0.337
	HID	3.073	0.409-23.097	0.275
Drugs used for GVHD prophylaxis	cyclosporine/tacrolimus	1.526	0.458-5.086	0.491
MNC		1.001	0.926-1.083	0.975
CD34		1.019	0.878-1.182	0.808
Grade II-IV GVHD	no/yes	3.125	1.39-7.029	0.006
Conditioning regimen	MAC/RIC	0.860	0.395-1.874	0.705
Letermovir	no/yes	0.112	0.034-0.374	0.000

**Table 4:** Multivariate Cox regression of csCMVi 100 days after HSCT.

Variable	Subcategory	HR	95% CI	P-value
Grade II-IV GVHD	no/yes	2.173	0.96-4.922	0.063
Letermovir	no/yes	0.127	0.038-0.429	0.001

### 3.5 Immune reconstitution

Analyses of T-lymphocyte and B-lymphocyte subsets showed no significant differences between the letermovir and control groups in regard to CD3+, CD4+, CD8+, and CD19+ cell counts before transplantation (p=0.218, 0.485, 0.174, and 0.050, respectively). Ninety days, 180 days, and 1 year after transplantation, the counts of these cell subsets were significantly smaller in the letermovir group than in the control group (all p < 0.05,**Supplementary Table 1**).

**Figure 2:**
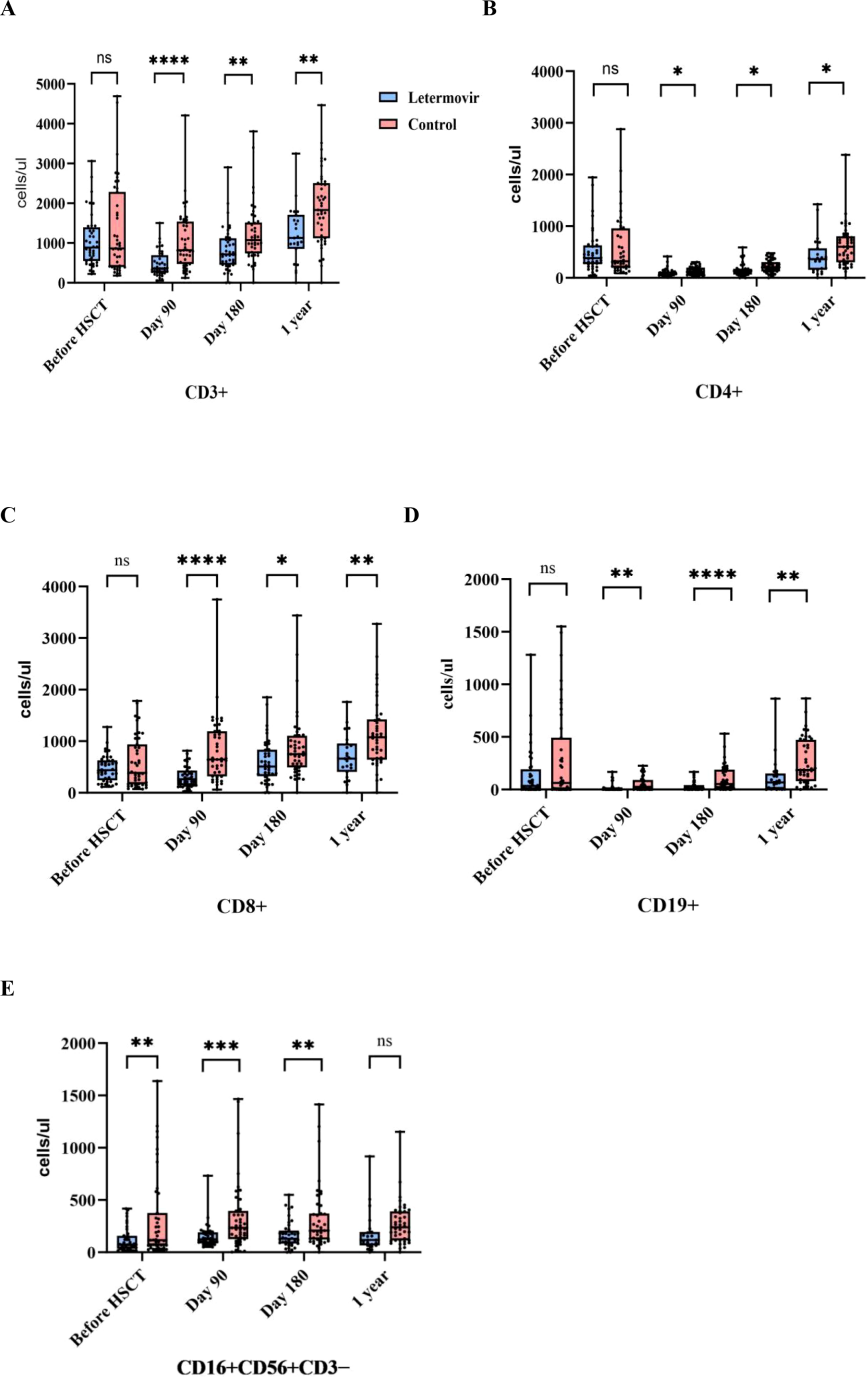
Analysis of immune function before and after transplantation. (**A–D**) There was no difference in CD3+, CD4+, CD8+, and CD19+ cell counts before transplantation. After transplantation, the counts in the letermovir group were lower than those in the control group.**(E)**CD16+CD56+CD3− cell counts were lower in the letermovir group than in the control group before transplantation and at 90 and 180 days after transplantation, but there was no difference between the two groups at one year after transplantation… ns, not significant; *p ≤ 0.05, **p ≤ 0.01, ***p ≤ 0.001, ****p ≤ 0.0001.

Analysis of NK cells showed significant differences between the two groups in the CD16+CD56+/CD3- cell counts before transplantation and 90 and 180 days after transplantation (all p < 0.05,**Supplementary Table 1**), but the difference disappeared by 1 year after transplantation (p=0.094; **Figure 2**).

**Figure 3:**
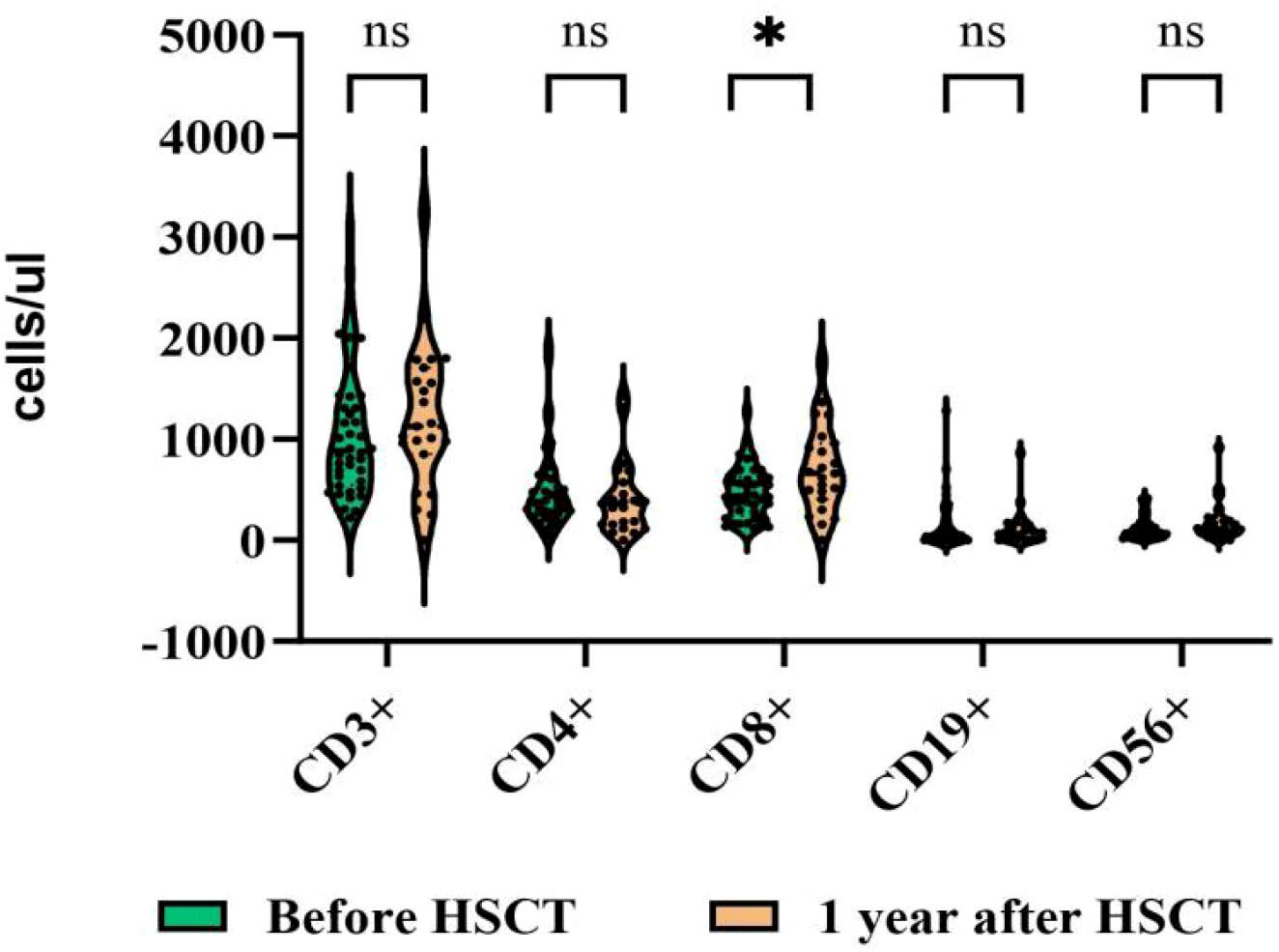
One year after transplantation, CD3+, CD4+, CD19+, and CD16+CD56+CD3− cell counts in the letermovir group showed no difference compared to pre-transplantation, while the CD8+ cell count was significantly higher. ns, not significant; *p ≤ 0.05.

Further analysis of the dynamic changes in immune reconstitution within the letermovir group revealed that, 1 year after transplantation, there were no significant differences in the CD3+, CD4+, CD19+, or CD16+CD56+/CD3- cell counts compared with pre-transplantation (p=0.397, 0.382, 0.716, and 0.134, respectively). In contrast, the CD8^+^ cell count was significantly higher (703.61 ± 430.57 vs. 473.85 ± 259.02 pre-transplantation, p=0.027; **Figure 3**). Notably, the CD16+CD56+/CD3- cell count in this group had already returned to the pre-transplantation level by 90 days after transplantation (150.07 ± 113.94 vs 120.55 ± 115.53,p=0.253),and the CD8+ cell count had returned to the pre-transplantation level by 180 days (603.43 ± 429.39 vs. 473.85 ± 259.02, p=0.107).

## 4 Discussion

This study retrospectively analyzed the efficacy and safety of letermovir for primary CMV prophylaxis in pediatric allo-HSCT recipients and its impact on immune reconstitution, comparing outcomes with a historical control group that did not receive letermovir prophylaxis. The cumulative incidence of CMV viremia 100 and 180 days post-transplantation was significantly lower in the letermovir prophylaxis group. These findings are consistent with multiple recent pediatric studies (12–15), confirming the significant preventive effect of letermovir in the pediatric population. A systematic review and meta-analysis published in 2025 indicated that letermovir prophylaxis can reduce the risk of CMV infection by approximately 70% in pediatric HSCT recipients (16). Particularly noteworthy are the latest data from a phase IIb pharmacokinetic study (P030) covering neonates to adolescents, which demonstrated that, when drug exposure levels comparable to those in adults are achieved, the efficacy of letermovir in preventing clinically significant CMV infection in children is similar to the efficacy in adults (17). This provides important evidence for the rational use of the drug in pediatric patients.

Letermovir plays an active role in improving the clinical course of CMV infection. In the present study, the mean time to CMV infection was significantly longer in the letermovir group than in the control group, with a shorter mean infection duration and a trend toward lower peak viral loads. A prospective study on umbilical cord blood transplant recipients in 2023 noted that, although approximately half of the patients could reconstitute CMV-specific cell-mediated immunity while receiving letermovir, the response intensity was generally weak and the drug did not fully prevent CMV reactivation after discontinuation (18). Similarly, we observed CMV reactivation in three pediatric patients after discontinuation (days 108–168). These findings suggest that letermovir may, through limited antigen exposure, “train” the host immune system to some extent, thereby delaying the occurrence of the first breakthrough infection and potentially making subsequent infections easier to manage (18, 19). However, both adult and pediatric studies indicate that the period after discontinuation (particularly between days 90 and 180) represents a risk window for CMV reactivation (17, 20), underscoring the necessity of extended monitoring and the implementation of individualized management strategies.

Letermovir demonstrated a favorable safety profile in pediatric patients. No significant differences were observed between the letermovir group and the control group regarding the time to neutrophil and platelet engraftment, the incidence of grade II-IV and III-IV aGVHD, impaired liver function, or the rates of EBV and BKV infection. Furthermore, no patients discontinued the drug due to treatment-related adverse events. These findings align with recently published safety data. A 2024 real-world study from Italy reported that the primary adverse events during letermovir prophylaxis in pediatric patients were gastrointestinal reactions, with no observations of significant myelosuppression or nephrotoxicity, indicating a superior safety profile compared to ganciclovir (21). In addition, pharmacokinetic studies focusing on adolescent populations have identified vomiting and fever as the most common adverse events, both of which are consistent with the typical spectrum of post-transplant complications, with no new safety signals detected (17).

Regarding EBV and BKV infections, although the infection rates in the letermovir group were numerically lower than those in the control group in this study, the differences did not reach significance. The mechanism of action of letermovir is highly specific to the CMV terminase complex and theoretically does not directly inhibit other herpesviruses, such as EBV and BKV. These findings are consistent with the theoretical expectation, indicating that the prophylactic effect of letermovir is virus-specific and does not interfere with the natural course of other herpesviruses. This further supports its favorable selective safety in clinical applications, which is particularly crucial for pediatric transplant patients whose immune systems are not yet fully developed and often face the risk of multiple viral infections. A 2025 study similarly noted that letermovir prophylaxis did not increase the rate of EBV reactivation and helped reduce the occurrence of co-infections with multiple viruses (22).

Univariate COX regression analysis indicated that both grade II-IV aGVHD and the absence of letermovir prophylaxis were risk factors for CMV infection within 100 days post-transplantation. In the multivariate analysis, the absence of letermovir prophylaxis remained an independent and significant risk factor, confirming the key protective role of letermovir in pediatric patients. On the other hand, the significance of grade II-IV aGVHD was attenuated in the multivariate analysis, which aligns with the current understanding of the underlying risk mechanism of aGVHD primarily increasing CMV risk through the intensified immunosuppressive therapy required for its treatment (23). Experiences from multiple pediatric centers also indicate that active aGVHD is an important risk factor for breakthrough CMV infection and reactivation. Effective prophylaxis with letermovir may partially mitigate this risk, suggesting that implementing prophylaxis in children with high-risk features, such as aGVHD, holds significant clinical importance (14, 24).

An important finding of this study is the detailed information on immune reconstitution in pediatric patients following letermovir prophylaxis. The results suggest that letermovir may delay the immune reconstitution process of T and B lymphocytes to some extent after transplantation. However, the mechanism underlying this phenomenon warrants further exploration. The traditional view holds that continuous exposure to viral antigens is a key signal driving the proliferation and differentiation of antigen-specific T cells. By effectively inhibiting CMV replication, letermovir significantly reduces immune system stimulation by CMV antigens, which may indirectly delay the expansion of CMV-specific T cell clones (19). A similar phenomenon has been observed during ganciclovir prophylaxis, in which potent viral suppression may be accompanied by delayed immune reconstitution (25). The lower T cell counts in the letermovir group during the early post-transplant period (e.g., 90 days) in this study support this hypothesis. A 2023 study in adults also reported that patients receiving letermovir prophylaxis exhibited slower and weaker reconstitution of CMV-specific T cell immunity compared to those receiving preemptive therapy (18). However, does this delay in immune reconstitution carry clinical significance? In this study, the incidence of other viral infections, such as EBV and BKV, and aGVHD was not significantly higher in the letermovir group, suggesting that, despite lower lymphocyte counts, the immune function in these patients may still be sufficient to control other opportunistic infections. Notably, In the letermovir group, NK cell (CD16^+^CD56^+^CD3^-^) counts were significantly lower than those in the control group before transplantation, but no significant difference was observed between the two groups at one year post-transplantation. Moreover, NK cell counts in the letermovir group returned to pre-transplantation levels by day 90 post-transplantation. NK cells are among the earliest lymphocyte subsets to reconstitute after hematopoietic stem cell transplantation, typically beginning to recover within 2–4 weeks post-transplantation. The recovery to baseline levels by day 90 observed in this study is generally consistent with the reconstitution kinetics reported in the literature (26). Therefore, the present study does not provide sufficient evidence to suggest that letermovir affects innate immune cell reconstitution. Recent studies have also suggested that NK cells play an important role in controlling CMV infection, and their earlier recovery may partially compensate for the temporary delay in T cell function (27). Notably, despite the lower baseline NK cell counts in the letermovir group, this group still demonstrated a significantly lower incidence of CMV infection, further supporting the protective effect of letermovir. In addition, B cell counts in the letermovir group remained persistently lower. This phenomenon may be related to changes in the immune microenvironment required for B cell activation following the suppression of CMV replication. The possibility of non-targeted regulatory effects of letermovir on the immune system cannot be entirely ruled out. CMV infection itself can profoundly reshape the B cell repertoire and influence humoral immune responses (28, 29). Therefore, effective suppression of CMV may alter the dynamic process of B cell reconstitution. However, the precise mechanisms remain to be elucidated.

By 1 year post-transplantation, the cellular immune function indicators in the letermovir group had recovered to pre-transplant levels. Particularly notable is the change in CD8+ T cells, as their counts were comparable to pre-transplant by 180 days post-transplantation and were significantly higher than pre-transplant levels by 1 year. As cytotoxic T lymphocytes, CD8+ T cells can directly recognize and eliminate infected cells, playing a crucial role in controlling primary infection and preventing CMV reactivation. Existing studies have shown that the presence of CMV-specific CD8+ T cells is closely associated with protective efficacy against CMV infection and related diseases (30).

Overall, the immunological data from this study reveal an important clinical balance: While letermovir is highly effective in preventing CMV infection, its use may be accompanied by a temporary delay in adaptive immune reconstitution. This phenomenon is particularly noteworthy in pediatric patients, whose immune systems are still developing, because the speed and quality of post-transplant immune reconstitution directly impact long-term survival and quality of life (31). Thus, whether dynamic monitoring of CMV-specific immune function (e.g., Quantiferon-CMV, ELISPOT, etc.) can enable individualized adjustment of the duration of prophylaxis has become a crucial direction for precise clinical management (32). Currently, a prospective study aiming to evaluate the value of CMV-specific immune reconstitution in predicting the risk of late infection after letermovir discontinuation is underway: INMUNOEND (NCT06814301). The design of this study stems from the aforementioned scientific question and provides an evidence-based exploratory pathway for future individualized prophylaxis strategies in pediatric patients (33).

Several limitations should be acknowledged. First, the use of a historical control group may introduce temporal bias, as patients in the control and letermovir groups were treated in different time periods. To mitigate this concern, key study parameters, including CMV monitoring frequency, preemptive therapy initiation criteria, and the antiviral agents used for preemptive therapy, remained consistent throughout the study period. However, unmeasured improvements in supportive care, clinical experience, or concomitant medication management over time may still have influenced the observed outcomes. Additionally, this study was limited by its single-center, retrospective design and relatively small sample size. The absence of CMV-specific immune functional assays also precluded a more in-depth assessment of immune reconstitution. Thus, the estimated treatment effect should be interpreted with caution.

This retrospective study in pediatric patients confirmed that the use of letermovir for primary cytomegalovirus (CMV) prophylaxis after allogeneic hematopoietic stem cell transplantation significantly reduced the incidence of CMV infection, improved the clinical course of the infection, and demonstrated a favorable safety profile. Multivariate analysis further established letermovir as an independent protective factor against early post-transplant CMV infection. Additionally, this study provides the first indication that letermovir may delay the numerical reconstitution of T cells, and B cells after pediatric transplantation. However, during the one-year follow-up period, this delay did not translate into a significant increase in clinical adverse events. Future multicenter, prospective, randomized controlled trials, incorporating more comprehensive immunological evaluations, are necessary to further validate the long-term efficacy and immunological effects of letermovir in pediatric patients. Such efforts would provide stronger evidence to optimize clinical practice and improve the overall prognosis for children undergoing transplantation.

## Data Availability

The original contributions presented in the study are included in the article/**Supplementary Material**. Further inquiries can be directed to the corresponding authors.

## Supplementary material

Supplementary Table 1 Mean lymphocyte subset counts in the letermovir and control group.
