# Supplementary material for: Clinical and immunological analysis of letermovir for preventing cytomegalovirus infection in children after hematopoietic stem cell transplantation
Source: Front Immunol. 2026 Apr 22;17:1817638. doi: 10.3389/fimmu.2026.1817638 (PMC13143763; doi:10.3389/fimmu.2026.1817638)
Supplement: Supplementary Table 1 — Mean lymphocyte subset counts in the letermovir and control group. [file Table1.pdf]

Supplemental Table 1. Mean lymphocyte subset counts in the letermovir and control group

**Immune cell subtype**	**Time**	**Letermovir mean±SD,cells/ul**	**Control mean±SD,cells/ul**	**t-value**	**p-value**
CD3+					
	Before HSCT	1067.6±665	1321.4±1145.91	-1.244	0.218
	day 90	472.48±318.84	1052.56±754.86	-4.616	0.000
	day 180	834.35±564.68	1222.23±742.37	-2.644	0.009
	1 year	1220.57±721..04	1907.58±925.84	-3.089	0.003
CD4+					
	before HSCT	508.25±424.11	587.53±595.38	-0.702	0.485
	day 90	100.53±77.71	139.7±76.71	-2.311	0.023
	day 180	153.68±124.22	222.93±117.34	-2.612	0.011
	1 year	414.96±368.34	623.40±411.85	-2.03	0.046
CD8+					
	before HSCT	473.85±259.02	591.84±494.76	-1.374	0.174
	day 90	310.45±203.08	820.12±647.04	-4.912	0.000
	day 180	603.43±429.39	884.09±639.46	-2.330	0.022
	1 year	703.61±430.57	1145.47±670.23	-2.857	0.006
CD19+					
	before HSCT	141.98±249.19	297.02±437.85	-2	0.05
	day 90	17.56±38.74	55.72±68.27	-3.158	0.002
	day 180	28.78±45.23	104.47±120.70	-3.833	0.000
	1 year	120.09±186.46	264.23±218.82	-2.813	0.007
CD16+CD56+CD3-					
	before HSCT	120.55±115.53	314.09±403.54	-3.015	0.004
	day 90	150.07±113.94	334.30±334.61	-3.404	0.001
	day 180	165.23±128.83	313.35±304.64	-2.920	0.005
	1 year	181.83±203.75	271.05±204.10	-1.701	0.094
